# Platelet aggregation rate serves as a significant predictive indicator for thromboembolic events in the context of stent-assisted embolization for unruptured arterial aneurysms

**DOI:** 10.3389/fneur.2025.1538753

**Published:** 2025-05-01

**Authors:** Xiaopeng Huang, Tingbao Zhang, Yu Feng, Xiang Li, Kui Liu, Wenyuan Zhao

**Affiliations:** ^1^Department of Neurosurgery, Zhongnan Hospital of Wuhan University, Wuhan, China; ^2^Brain Research Center, Zhongnan Hospital of Wuhan University, Wuhan, China; ^3^Frontier Science Center for Immunology and Metabolism, Wuhan University, Wuhan, China; ^4^Medical Research Institute, Wuhan University, Wuhan, China; ^5^Sino-Italian Ascula Brain Science Joint Laboratory, Wuhan University, Wuhan, China

**Keywords:** platelet aggregation rate, stent-assisted embolization, unruptured intracranial aneurysms, thromboembolic events, antiplatelet therapy

## Abstract

**Background:**

Perioperative cerebrovascular thromboembolic events are serious complications of stent-assisted embolization (SAE) for unruptured intracranial aneurysms (UIAs). To date, there have been no definitive clinical trial results to effectively predict and prevent the occurrence of this complication. This study aims to elucidate the correlation between platelet aggregation rate (PAR) and thromboembolic events (TEs), with the goal of predicting the occurrence of cerebrovascular TEs in these patients.

**Methods:**

In this retrospective, single-center cohort study, we included 704 cases of unruptured intracranial aneurysms treated with stent-assisted intervention from 2016 to 2020. Cerebrovascular TEs were defined as cerebral ischemic events occurring within 7 days before or after the interventional procedure. Light Transmission Aggregometry (LTA) was used to detect PAR in patients. Clinical data, including patients’ demographic information and perioperative PAR, were collected. Multivariate analysis was conducted to examine the correlation between these factors and the occurrence of TEs. Additionally, Lasso regression was employed to select clinical indicators associated with perioperative TEs. Receiver Operating Characteristic (ROC) curves were generated for prognostic indicators such as PAR, with the optimal cutoff value determined. A nomogram was then simulated, and predictive accuracy of the model was evaluated using Decision Curve Analysis (DCA).

**Results:**

A total of 562 patients were included in the final analysis. Significant differences were observed in the incidence of thrombosis between the control group and the experimental group (9.38% vs. 4.96%). The ROC curve of platelet aggregation index, highly correlated with prognosis and derived from Lasso regression, identified the optimal cutoff value for the maximum preoperative PAR as 19.81. A nomogram was constructed based on selected clinical baseline data, and its calibration was assessed using data from the prediction group. The net benefit of the experimental group model’s DCA curve was significantly improved.

**Conclusion:**

For patients undergoing SAE for UIAs, utilizing PAR and other indicators as reference standards for treatment results in better prognosis compared to empirical treatment based on guidelines. Guiding antiplatelet therapy using PAR and other indicators is both meaningful and beneficial to clinical practice.

## Introduction

With the continuous advancement in equipment and technology, numerous modalities have been developed for unruptured intracranial aneurysms (UIAs) (1). Endovascular embolization of aneurysms, recognized for its relatively lower risks and reduced bleeding, has gained widespread application (1–3). In recent years, the introduction of stent-assisted techniques has significantly improved the prognosis of wide-necked and complex aneurysms (2). However, one of the serious complications for stent-assisted embolization (SAE) is the occurrence of arterial thromboembolic events (TEs), which can lead to permanent neurological deficits, making this a severe complication of the treatment (4–7).

In clinical practice, dual antiplatelet therapy (DAPT) with the oral administration of 100 mg aspirin and 75 mg clopidogrel is commonly recommended by guidelines to prevent such complications (8–11). Previous studies on DAPT have primarily focused on cardiac interventions, with relatively few addressing SAE for UIAs (12–15). Consequently, there is a lack of precise examination criteria to guide medication protocols, leading to the persistent occurrence of perioperative TEs. Various factors, including low platelet function and clopidogrel resistance, affect the efficacy of DAPT (16–18). The CHANCE-2 large-sample clinical controlled trial indicated a significant decrease in the antiplatelet effect of clopidogrel in carriers of the *CYP2C19* loss-of-function allele (10, 19–22). It is generally accepted that platelet function testing can evaluate the efficacy of DAPT and the occurrence of TEs (8, 23, 24).

Verification methods such as VerifyNow, Light Transmission Aggregometry (LTA), TEG (22, 25–27) are commonly used to determine platelet function. Previous studies have demonstrated the efficacy of the VerifyNow testing method (28). Guidelines suggest using PAR as one of the indicators for prognosis assessment. However, there is currently no clear correlation analysis between PAR measured by LTA and the occurrence of perioperative TEs (29–31). Additionally, there has not been a detailed prognosis model developed based on this examination to predict outcomes.

Therefore, this study aims to further investigate the impact of LTA PAR testing on the occurrence of perioperative TEs in SAE for UIAs. We seek to construct a clinical prognosis model that can provide decision-making basis for DAPT during the perioperative period in clinical practice.

## Methods

### Source of cases

Our center is a major medical institution for the treatment of cerebrovascular diseases in the central region of China, equipped with a comprehensive database containing a large number of cerebrovascular disease cases, clinical data, and sophisticated examination and treatment methods. The samples for this study are all based on data in this database. We collected data on UIAs treated with SAE from 2016 to 2020, incorporating four different stent devices: the LEO, LVIS, Enterprise, and Neuroform Atlas. Exclusion criteria included intraoperative aneurysm rupture, pregnancy, other hypercoagulable states, severe systemic diseases, hematologic disorders, use of flow-diverter and patients with a history of antiplatelet medication use. All patients were divided into three groups based on conditions. The control group consisted of patients who received empirical treatment without platelet aggregation testing. The experimental and prediction groups comprised patients who underwent surgery based on platelet aggregation rate (PAR) as an indicator (generally considered achieving a PAR below 25%). After statistical analysis, the cases were randomly assigned to the experimental and prediction groups.

### Stent-assisted embolization treatment and complications

To reduce the occurrence of TEs we routinely administer 75 mg of clopidogrel and 100 mg of aspirin orally each day for 7 days prior to surgery and continue this DAPT for 1 month postoperatively. For patients who exhibit clopidogrel resistance identified through *CYP2C19* genetic testing, we replace clopidogrel with ticagrelor (*D* = 86) for treatment. These patients are excluded from our study. We perform routine digital subtraction angiography (DSA) before surgery to confirm the status of the aneurysm and blood vessels, ensuring the success of the procedure. A PAR below 25% within 24 h before surgery is considered a criterion for proceeding with the operation.

Interventional surgical approach: All surgeries are performed under general anesthesia with the patient in a supine position and systemic continuous heparinization was administered intraoperatively. The protocol was as follows: heparinization was administered according to the patient’s body weight, with an initial dose of 200/3 U/kg, followed by an additional 1/2 of the previous dose every hour until a final dose of 1,000 U/h was reached, and postoperative heparinization was neutralized by natural neutralization. During the procedure, bilateral femoral arteries were punctured using the Seldinger technique. One femoral artery was used for angiography to localize the aneurysm and assess intraoperative thrombosis, while the other femoral artery was utilized for guidewire-guided SAE. Stent-assisted techniques are generally employed for wide-necked aneurysms and aneurysms in complex locations to achieve satisfactory embolization results. After stent implantation, X-per CT examination was performed to observe whether there was intracranial bleeding. Repeat imaging showed that the aneurysm was well embolized or contrast agent retention, smooth blood flow in the main and branch arteries of the parent artery, and good stent morphology. After surgery, patients undergo routine DAPT and hematologic parameter monitoring. Postoperatively, patients are transferred to the Neuro-Intensive Care Unit (NICU) for 1 day of intensive monitoring and then transferred to a regular ward. Follow-up imaging examinations are promptly conducted postoperatively to evaluate for hemorrhage and infarction.

### Thromboembolic events

Cerebrovascular infarction events are defined as neurological symptoms occurring within 7 days after surgery, such as:

1. Weakness or numbness on one side of the body (with or without facial involvement).
2. Facial numbness or drooping of the mouth on one side.
3. Slurred speech or difficulty understanding language.
4. Gaze deviation to one side or bilateral.
5. Loss or blurred vision on one side or both eyes.
6. Dizziness accompanied by vomiting.
7. Previously rare severe headache or vomiting.
8. Altered consciousness or seizures.

Or, based on CT or MRI findings suggestive of thrombotic events. CT scans are used to rule out intracerebral hemorrhage (ICH), or CT angiography shows enhanced intracranial arteries reflecting intracranial arterial infarction (lasting more than 24 h). MRI findings include normal signal intensity on diffusion-weighted imaging (DWI, *b* = 0) and T2-weighted imaging (T2WI), and high signal intensity on DWI (*b* = 1,000), indicating fresh infarct lesions.

### Platelet aggregation rate testing

In our center, we use LTA to measure the PAR. Specifically, platelet-rich plasma (PRP) is placed in a cuvette, and various agonists (such as ADP, adrenaline, collagen, thrombin, arachidonic acid, TXA2, PAF, etc.) are added. The mixture is stirred with a silicon-coated magnetic bead, causing gradual platelet aggregation, resulting in decreased plasma turbidity and increased light transmission. This change is recorded to generate a dynamic curve of platelet aggregation. The aggregation rate and light transmission of PRP are defined as 0%, while the aggregation rate and light transmission of platelet-poor plasma (PPP) are defined as 100%. Platelet aggregation is automatically measured, recorded, and plotted using a platelet aggregometer. The PAR is determined with reference to 100 and 0% aggregation rates. After adding agonists to the sample, the proportion of change within 120 s compared to the reference system is recorded as the 120-s aggregation rate. Generally, the maximum change within 5 min compared to the reference system is considered the maximum aggregation rate for the sample. PAR measurements are conducted within 24 h before surgery for preoperative samples and within 3 days after surgery for postoperative samples.

### CYP2C19 gene typing

Using a human *CYP2C19* gene typing test kit, we identify mutations at the *CYP2C19* allele loci *CYP2C192* (681G → A, rs4244285), *CYP2C193* (636G → A, rs4986893), and CYP2C19*17(−806C → T, rs12248560). Based on the combination of mutation alleles at these loci, patients are categorized into different *CYP2C19* enzyme metabolic types: poor metabolizers (*2/*2, *2/*3, or *3/*3) and intermediate metabolizers (*1/*2 or *1/*3).

### Data collection and processing

Patients’ baseline clinical data include age, gender, hypertension, diabetes, smoking status, alcohol consumption, aneurysm neck size, aneurysm dome diameter, location, platelet laboratory parameters, and lipid laboratory parameters. These data are obtained from our center’s database. Hypertension is defined as a history of systolic blood pressure >140 mmHg or diastolic blood pressure >90 mmHg on previous examinations. Hyperlipidemia is defined as a history of diagnosed hyperlipidemia, plasma total cholesterol concentration >5.17 mmol/L, or plasma triglyceride concentration >2.3 mmol/L. Smoking and alcohol consumption are defined as a history of smoking/alcohol consumption for more than 1 year and not quitting. Aneurysm size includes measurements of the aneurysm neck and body, and aneurysm location is categorized as anterior circulation of the brain, internal carotid artery, middle and posterior cerebral artery, or posterior circulation of the brain. Aneurysm measurements are obtained from imaging studies following DSA.

### Plotting graphs

#### Clinical baseline data table

We used the “tableone” package to represent categorical variables (gender, smoking, alcohol consumption, hypertension, hyperlipidemia, aneurysm location, occurrence of stroke events) as counts or percentages. Continuous variables with a normal distribution (platelet laboratory parameters, lipid laboratory parameters) were represented as mean ± standard deviation, while those with a non-normal distribution (age) were represented as median and interquartile range. All other settings were left as default.

#### ROC curve

We used the “pROC” package, with the outcome set as “occurrence of stroke events,” to generate the ROC curve. We also displayed the AUC and added the cutoff points. All other settings were left as default.

#### Lasso regression

We used the “glmnet” package to convert the data table into matrix form and build the model. We utilized cross-validation with the “cv.glmnet” function to select the optimal lambda and obtain the final model under this lambda coefficient. All other settings were left as default.

#### Nomogram alignment diagram

Based on the final model selected through Lasso regression, we utilized the “rms” package to compute the column line table, and then used the “rms::calibrate” function to plot the calibration curve. All other settings were left as default.

#### DCA plot

Using the “rmda” package, we fitted the selected clinical model after screening through logistic regression as a single curve, with the outcome variable set as “occurrence of TEs.” All other settings were left as default.

### Statistical analysis

We evaluated the normality of sample distributions using the Shapiro–Wilk test. Normally distributed continuous data were reported as mean (±SD) and analyzed using the *t*-test. Non-normally distributed continuous data were expressed as median and interquartile range, and analyzed using the Mann–Whitney *U* test. Count data were presented as percentages and analyzed using the chi-square test and Fisher’s exact tests. Logistic and Cox regression analyses were employed to analyze independent related factors. The statistical significance level was defined as *p* < 0.05 (*), *p* < 0.01 (**), *p* < 0.001 (***), *p* < 0.0001 (****). Data processing was conducted using SPSS V26.0 and R.

## Results

### Using PAR as a guide for DAPT helps reduce the incidence of stroke

We compared the clinical baseline data tables of the control group (96 cases) and the experimental group (363 cases) (Tables 1, 2). Based on our data comparison, there were no significant differences in patient gender, age, underlying diseases, etc., between the two groups regarding the occurrence of stroke events. However, a statistically significant difference in the indicator of PAR was observed between the two groups regarding the occurrence of stroke events. Additionally, we compared the proportions of perioperative stroke events between the control and experimental groups (9.38% vs. 4.96%), and found improved outcomes in the latter. Our clinical practice and data analysis indicate that guiding perioperative DAPT for UIAs assisted by stent intervention based on PAR testing can improve patient outcomes.

**Table 1:** Baseline information of control group.

Factors	Total (*n* = 96)	TEs (*n* = 9)	Non-TEs (*n* = 87)	*p*-value
Age, median [IQR], years	55.5 (40.25, 73.75)	50 (39.5, 81)	57 (40, 73)	0.6315
Man, *n* (%)	56 (58.33%)	9 (100%)	47 (54.02%)	0.0094**
Smoking, *n* (%)	48 (75.10%)	2 (22.22%)	46 (52.87%)	0.1586
Drinking, *n* (%)	48 (50.00%)	4 (44.44%)	44 (50.57%)	>0.9999
Hypertension, *n* (%)	40 (41.67%)	3 (33.33%)	37 (42.53%)	0.7308
Hyperlipidaemia, *n* (%)	44 (45.83%)	5 (55.56%)	39 (44.83%)	0.7279
Position				0.4378
ICA (C1-C5), *n* (%)	21 (21.88%)	2 (22.2%)	19 (21.84%)	
ICA (C6-C7), *n* (%)	20 (20.83%)	1(11.11%)	19 (21.84%)	
Anterior circulation + MCA, *n* (%)	16 (16.67%)	0	16 (18.39%)	
PcoA, *n* (%)	19 (19.79%)	3 (33.33%)	16 (18.39%)	
Posterior circulation, *n* (%)	21 (21.88%)	3 (33.33%)	17 (19.54%)	
Dome size, median [SEM], mm	6.03 (0.25)	6.30 (0.77)	6.00 (0.26)	0.7317
Neck size, median [SEM], mm	4.87 (0.20)	4.18 (0.59)	4.95 (0.21)	0.2649

*p < 0.05, **p < 0.01, ***p < 0.0001.

**Table 2:** Baseline information of experimental group.

Factors	Total (*n* = 363)	TEs (*n* = 18)	Non-TEs (*n* = 345)	*p*-value
Age, median [IQR], years	56.00 (46.00, 64.00)	60 (46.75, 64.00)	56.00 (45.50, 65.00)	0.5754
Man, *n* (%)	163 (44.90%)	7 (38.89%)	156 (45.22%)	0.6364
Smoking, *n* (%)	201 (55.37%)	6 (33.33%)	195 (56.52%)	0.0861
Drinking, *n* (%)	178 (49.04%)	11 (61.11%)	167 (48.41%)	0.3394
Hypertension, *n* (%)	193 (53.17%)	6 (33.33%)	187 (54.20%)	0.0944
Hyperlipidaemia, *n* (%)	195 (53.72%)	7 (38.89%)	188 (54.49%)	0.2299
Position				0.2740
ICA (C1-C5), *n* (%)	69 (19.01%)	3 (16.67%)	66 (19.13%)	
ICA (C6-C7), *n* (%)	84 (23.14%)	2 (11.11%)	82 (23.77%)	
Anterior circulation + MCA, *n* (%)	58 (16.00%)	1 (5.56%)	57 (16.52%)	
PcoA, *n* (%)	78 (21.49%)	7 (38.89%)	71 (20.58%)	
Position	74 (20.39%)	5 (27.78%)	69 (20.00%)	
prePAG120, median [SEM]	13.68 (0.27)	15.20 (1.27)	13.6 (0.27)	0.1987
prePAGmax, median [SEM]	17.29 (0.22)	21.81 (0.58)	17.05 (0.22)	<0.0001****
preADP test, median [SEM]	50.56 (1.51)	51.52 (6.98)	50.51 (1.55)	0.8843
preAA test, median [SEM]	52.22 (1.48)	52.11 (7.70)	52.22 (1.51)	0.9861
postPAG120, median [SEM]	16.48 (0.24)	17.22 (0.53)	16.44 (0.26)	0.4890
postPAGmax, median [SEM]	18.40 (0.25)	20.96 (0.43)	18.26 (0.26)	0.0171*
postADP test, median [SEM]	47.89 (1.55)	45.86 (7.73)	48 (1.58)	0.7657
postAA test, median [SEM]	49.08 (1.48)	69.75 (5.45)	48 (1.51)	0.0014**
Dome size, median [SEM], mm	5.893 (0.13)	5.76 (0.34)	5.9 (0.13)	0.8118
Neck size, median [SEM], mm	6.097 (0.12)	5.84 (0.47)	6.11 (0.12)	0.6121

*P < 0.05, **P < 0.01, ****P < 0.0001.

### Lasso regression was used to select clinical indicators strongly correlated with prognosis

**Figure 1:**
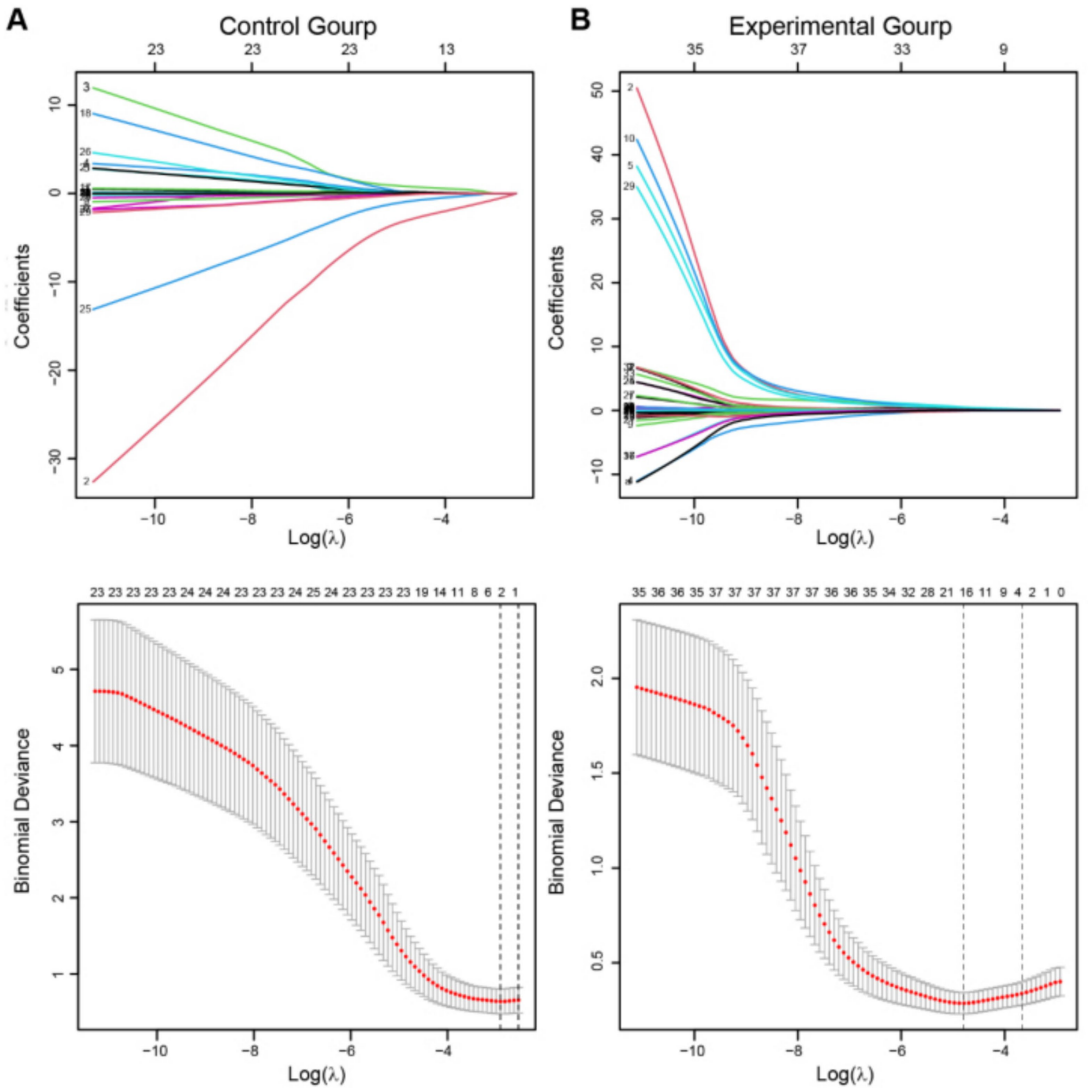
Lasso regression was employed to screen clinical variables. **(A,B)** The above figures depict the regression coefficient path plots for the experimental group and the control group, respectively. The below figures show the cross-validation curves for both the experimental and control groups. The dashed line on the left represents the minimum deviation *λ* value (λ. min), while the dashed line on the right represents one standard error to the right of λ min (λ. 1-s.e.). λ. 1-s.e. was adopt as the final equation selection criterion.

We aim to construct a model to predict the incidence of thrombosis in patients with UIAs undergoing embolization therapy, based on the collected clinical baseline data. Recognizing that various clinical indicators have different impacts on patient prognosis, we employed Lasso regression to select the most clinically relevant indicators. Initially, we used data from the experimental group to screen clinical baseline indicators, followed by Lasso analysis. An appropriate *λ* value was then selected based on cross-validation curves to determine the final model. Ultimately, we identified the following clinically relevant indicators strongly associated with thrombotic events: smoking, hypertension, hyperlipidemia (HLP), prothrombin time (PT), prothrombin activity (PTA), international normalized ratio (INR) in preoperative coagulation function tests, postoperative platelet count (PLT), D2 dimer (DD), triglycerides (TG), high-density lipoprotein (HDL), preoperative 120 s platelet aggregation rate (Pre 120 s PAR), maximum aggregation rate (Pre Max PAR), postoperative 120 s platelet aggregation rate (Post 120 s PAR), maximum aggregation rate (Post Max PAR), and maximum platelet aggregation rate induced by arachidonic acid (Post AA MAR) (Figure 1A).

The same screening process was subsequently carried out in the control group (Figure 1B). Based on the optimal *λ* value obtained, the final model revealed gender and postoperative fibrinogen (Post FIB) as clinically relevant indicators strongly associated with TEs in the control group.

Notably, by combining the results of Lasso regression from both the control group (Figure 1A) and the experimental group (Figure 1B), we included the PAR index for factor analysis. A significant correlation between PAR and the occurrence of TEs was discovered, in addition to the basic clinical baseline indicators. Eventually, five PAR indices were selected.

### PAR can serve as a clinical diagnostic indicator for assessing the efficacy of SAE in the treatment of UIAs

**Figure 2:**
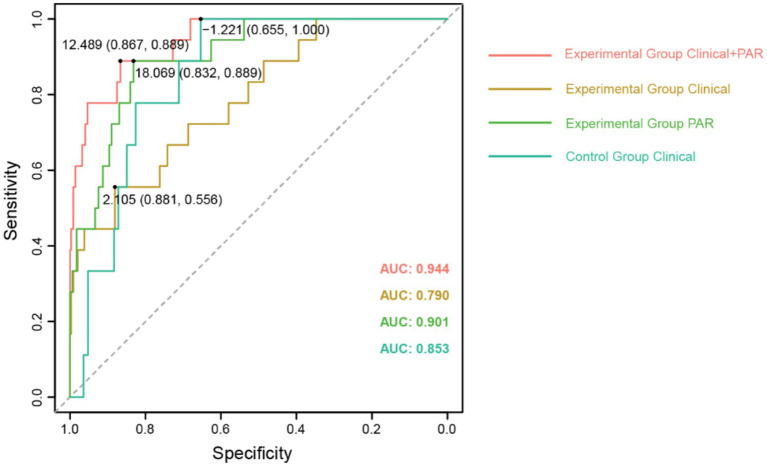
The comparison of ROC curves for fitting clinical baseline data. The ROC curves with AUC values for different fitting curves have been displayed, along with the cut-off values for each curve.

To further investigate these five PAR indices, logistic regression analysis was used to fit the filtered clinical baseline data, including: Clinical for the control group; Clinical, Clinical + PAR, and PAR for the experimental group. These represent four different fitting curves with varying variables. Here, PAR represents the fitting result of the five selected platelet aggregation indices, while Clinical represents the fitting result of other clinical indicators. We then calculated the ROC values for these four fitting curves. Comparison revealed that in the experimental group, Clinical + PAR had the largest area under the curve (AUC = 0.944, Figure 2), indicating the highest predictive performance for thrombotic events during the perioperative period. Additionally, the AUC for PAR in the experimental group was higher than that for Clinical alone (0.901 vs. 0.790, Figure 2), demonstrating significant improvement in predictive effectiveness for TEs when PAR indices were added to the clinical baseline data.

### The definition of the clinical threshold for PAR

**Figure 3:**
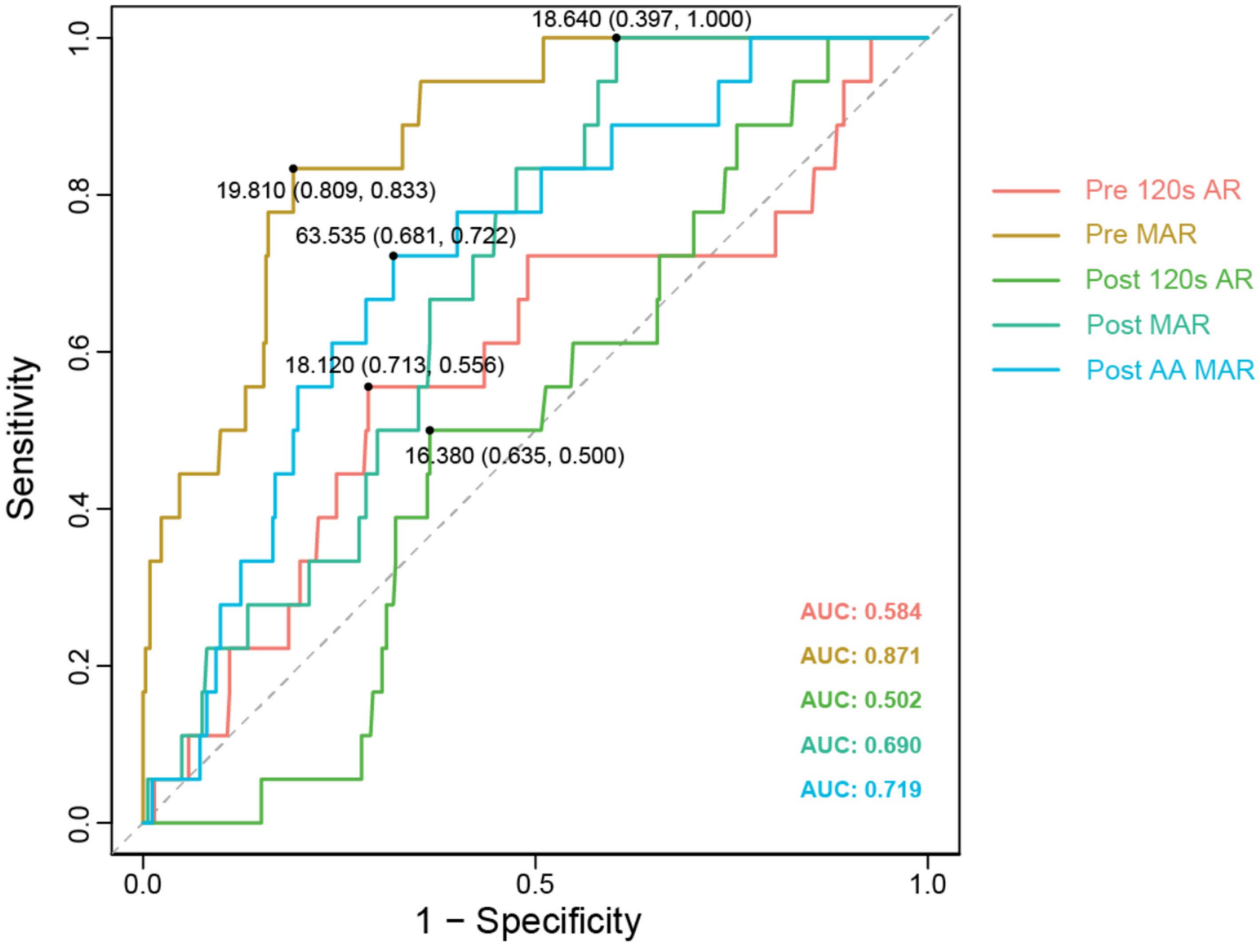
Different PAR test indicators ROC comparison. The ROC curves with AUC values for different fitting curves have been displayed, along with the cut-off values for each curve.

Subsequently, based on data from the experimental group, we calculated the optimal cutoff values for the ROC curves of each index in PAR, ultimately, determining the clinical threshold values for platelet aggregation. Notably, the AUC value for Pre MAR was significantly higher at 0.871 (Figure 3) compared to the other indices: AUC for Pre 120 s PAR was 0.584; Post 120 s PAR was 0.502 (Figure 3); Post MAR was 0.690 (Figure 3); and postoperative maximum aggregation rate induced by arachidonic acid was 0.719 (Figure 3). This indicates that utilizing the Pre MAR index for predicting TEs is more accurate. The optimal cutoff value corresponding to Pre MAR in the ROC was determined to be 19.81. To improve patient prognosis, we recommend DAPT during the perioperative period to ensure that the maximum preoperative PAR is below this threshold value.

### Construction of a clinical prediction model based on PAR

**Figure 4:**
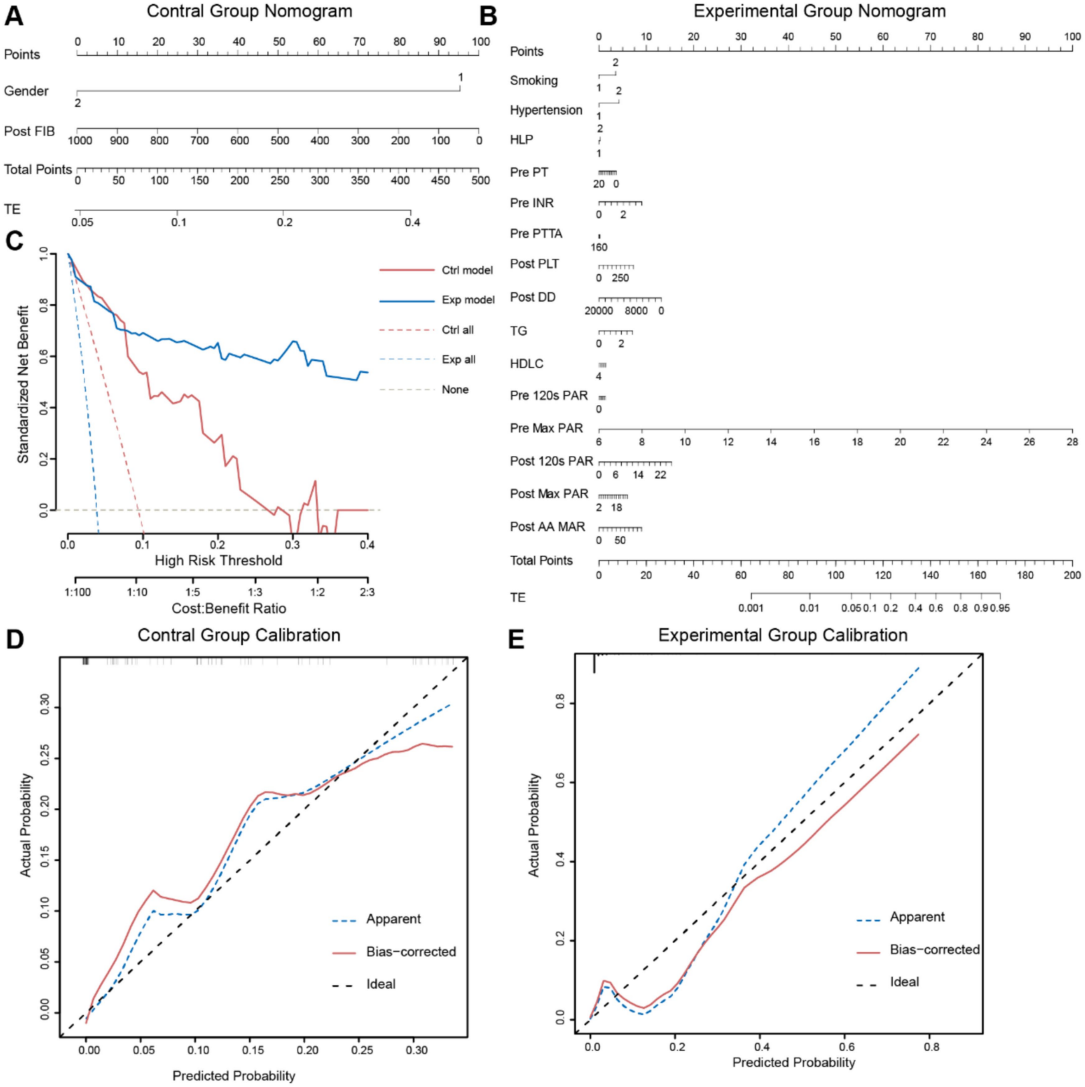
Two sets of data’s nomogram and internal validation. **(A,B)** Using the Nomogram to predict thrombotic events. The Nomogram provides a way to calculate the probability of thrombotic events using clinical baseline data. To utilize it, first, draw a vertical line on the gender axis (1: Male; 2: Female) perpendicular to the Point axis, and record the intersection point as the score for Gender. Then, similarly record scores on other axes, sum up the scores, compare them to the Total Points axis, and draw a vertical line from the corresponding point on this axis to the TE axis. The intersection point represents the probability of TE occurrence. **(C,D)** Calibration curves of the two groups validate the model. The goodness-of-fit is assessed using the Hosmer–Lemeshow test, with bootstrapping sampling (*n* = 100). **(E)** DCA plots of the two Nomograms. The *x*-axis represents the threshold probability, while the *y*-axis represents the net benefit. The white dashed line represents the net benefit without intervention, the blue and red dashed lines represent the net benefit values of interventions for all patients in the two groups, and the solid blue and red lines represent the net benefit values of interventions based on the predictive results of the two models.

**Figure 5:**
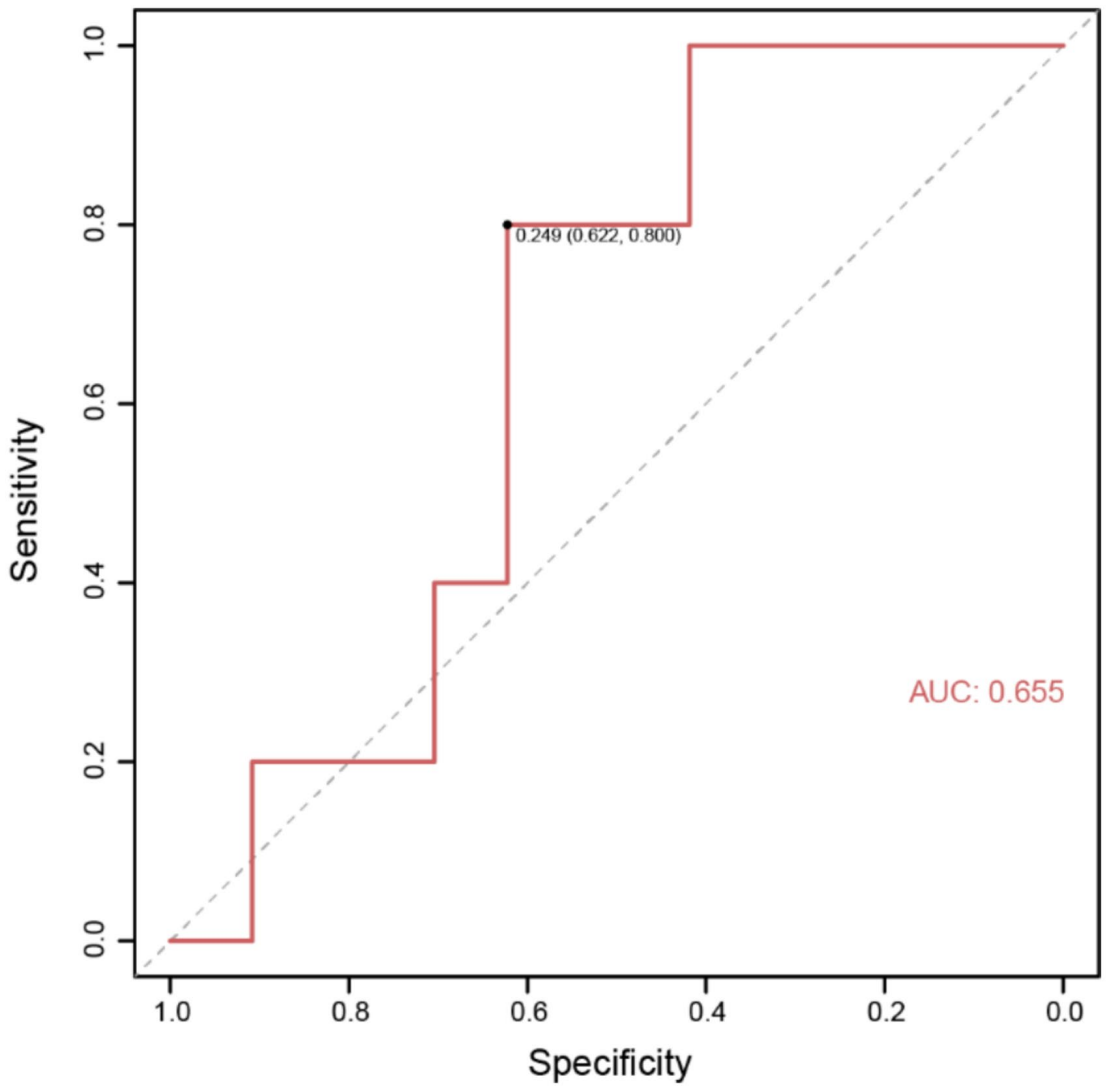
Receiver operating characteristic (ROC) of external data validation.

To better predict the occurrence of perioperative ischemic events in patients using clinical indicators, we constructed nomograms based on the clinical indicators selected by Lasso regression in the two groups (Figures 4A,B), along with corresponding calibration curves (Figures 4C,D). The *Z*-test yielded a *p*-value of 0.486, indicating satisfactory alignment between predicted values and actual values in the experimental group, with a correlation coefficient (Dxy) of 0.906, suggesting satisfactory fit for both curves. Drawing from decision curve analysis (DCA), we noted that the net benefit rate (NB) of the experimental group was significantly higher than that of the control group within the threshold probability range of 0–1 (Figure 4E). Subsequently, we validated the accuracy of the nomogram model in the experimental group using predictive data (Table 3), and the AUC of the ROC results indicated that the model had good prognostic ability (AUC = 0.655, Figure 5). Therefore, we believe that using PAR indicators to guide DAPT during the perioperative period of SAE for UIAs can help reduce the occurrence of perioperative ischemic events, which is clinically significant.

**Table 3:** Baseline information of prediction group.

Factors	Total (*n* = 103)	TEs (*n* = 5)	Non-TEs (*n* = 98)	*p*-value
Age, median [IQR], years	59 (48,73)	54.00 (49.5,62)	60(48,74)	0.5427
Man, *n* (%)	64 (62.14%)	5 (100%)	59 (60.20%)	0.1538
Smoking, *n* (%)	50 (48.54%)	3 (60.00%)	47 (48.00%)	0.6722
Drinking, *n* (%)	45 (43.70%)	1 (20.00%)	44 (44.90%)	0.3831
Hypertension, *n* (%)	49 (47.57%)	2 (40.00%)	47 (47.96%)	>0.9999
Hyperlipidaemia, *n* (%)	45 (43.70%)	4 (80.00%)	41 (41.84%)	0.165
Position				
ICA (C1-C5), *n* (%)	23 (22.33%)	0	23 (23.47%)	
ICA (C6-C7), *n* (%)	21 (20.39%)	3 (60.00%)	18 (18.37)	
Anterior circulation + MCA, *n* (%)	17 (16.50%)	0	17 (17.35)	
PcoA, *n* (%)	18 (17.48%)	1 (20.00%)	17 (17.35)	
Posterior circulation, *n* (%)	24 (23.30%)	1 (20.00%)	23 (23.47%)	
prePAG120, median [SEM]	12.03 (0.64)	21.17 (0.80)	11.56 (0.64)	0.001***
prePAGmax, median [SEM]	21.24 (0.74)	22.75 (0.48)	21.16 (0.77)	0.6461
preADP test, median [SEM]	55.05 (2.71)	48.07 (11.96)	55.4 (2.79)	0.5629
preAA test, median [SEM]	51.99 (2.90)	52.99 (16.71)	51.94 (2.95)	0.9379
postPAG120, median [SEM]	13.02 (0.61)	20.98 (1.38)	12.61 (0.61)	0.0028**
postPAGmax, median [SEM]	19.11 (0.56)	24.46 (1.77)	18.84 (0.57)	0.0309*
postADP test, median [SEM]	50.9 (2.76)	60.56 (8.86)	50.4 (2.86)	0.4312
postAA test, median [SEM]	51.55 (2.79)	55.65 (5.56)	51.34 (2.92)	0.7418
Dome size, median [SEM], mm	6.30 (0.20)	5.38 (1.25)	6.35 (0.20)	0.3034
Neck size, median [SEM], mm	6.77 (0.18)	6.51 (0.82)	6.79 (0.18)	0.7359

*P < 0.05, **P < 0.01, ***P < 0.001.

## Discussion

Treatment for unruptured aneurysms should be selected based on the characteristics of the aneurysm and individual patient preferences (1, 3, 30, 32). Currently, minimally invasive interventions, which offer less trauma, faster postoperative recovery, and fewer complications compared to open surgery, have gained broader acceptance among patients and their families. However, despite these advantages, there are still various challenges associated with interventional treatment of aneurysms (1, 4).

Traditionally, interventional treatment involves directly occluding the aneurysm with coil embolization, where coils are used to promote thrombosis within the aneurysm sac, thereby achieving closure of the aneurysm (1). However, due to variations in the neck-to-body ratio of aneurysms, there is a risk of coil protrusion in wide-necked aneurysms, which could lead to catastrophic consequences (6, 33, 34). To mitigate this risk, stent-assisted coil embolization is employed to ensure successful occlusion.

Stents, however, are foreign bodies within blood vessels, and their presence may trigger an immune response involving blood components, leading to platelets adhesion to the stent and subsequent thrombus formation. The detachment of these thrombi may result in cerebral infarction (34, 35). Therefore, controlling platelet adhesion to the stent through medication to prevent postoperative ischemic events is particularly important in stent-assisted coil embolization (1, 34).

In this study, four types of stents were included: LEO, LVIS, Enterprise, and Neuroform Atlas, with patients using flow-diverter devices excluded. According to the study by Kan et al. (36), the complication rates for the Enterprise, Neuroform Atlas, and LVIS stents were 10.5, 1.4, and 14.3%, respectively. Another meta-analysis indicated that the thromboembolic risk for the Neuroform Atlas stent was 2.9% (37). For the LEO stent, a study by Shen et al. (38) reported a TEs rate of 6.0% (5/83 patients), while another study involving 101 patients reported a rate of 8.7% (39). Furthermore, a meta-analysis on self-expanding braided stents showed that the thromboembolic complication rate for the LEO stent ranged from 2.5 to 28% (40). In the case of unruptured aneurysms, the overall complication rate in the LEO stent group was 9.6% (2/21), while no complications were observed in the Neuroform Atlas stent group. No significant statistical difference was found between the two groups (*p* > 0.05). In the study by Ge et al. (41), the TEs rate for the LVIS stent was 8.7% (8/92 patients), while for the Enterprise stent, it was 14.3% (14/98 patients). Although the complication rate for the Enterprise stent was higher than that for the LVIS stent, no significant difference was observed between the two. These findings suggest that different stent devices are associated with varying thromboembolic complication rates (41). In the case of unruptured aneurysms, the overall complication rate for the LEO stent group was 9.6% (2/21), while no complications were observed in the Neuroform Atlas stent group. No significant statistical difference was found between the two groups (*p* > 0.05).

The importance of antiplatelet therapy during the perioperative period has been widely recognized for its ability to effectively reduce TEs. Over-responders (with excessively low PAR due to over-inhibition) are more prone to bleeding complications, while non-responders (with insufficient platelet inhibition) face a higher risk of TEs. This finding is consistent with the research by Fifi et al. (9). Clopidogrel resistance is a trigger for ischemic complications during UIAs SAE (22, 42). Due to clopidogrel resistance, there is considerable variability in patients’ responses to antiplatelet medications. Non-responders receiving standard antiplatelet therapy still exhibit high PAR, putting them at a higher risk for thromboembolic complications, necessitating the use of alternative or adjusted antiplatelet therapies. On the other hand, over-responders may exhibit excessive platelet inhibition, increasing the risk of bleeding complications, and may require a reduction in the dosage of antiplatelet therapy to mitigate bleeding risks. Several studies have explored the relationship between PAR and bleeding complications, particularly in patients receiving DAPT. The study by Oxley et al. (8) emphasized that excessive platelet inhibition (low PAR) may make patients more susceptible to bleeding complications, especially in neurointerventional procedures, such as SAE. In contrast, the research by Choi et al. (6) indicated that clopidogrel resistance (high PAR) may increase the risk of TEs. PAR, especially when tested using LTA, can quantitatively assess platelet aggregation in response to various agonists, helping clinicians evaluate the efficacy of antiplatelet therapy.

Some studies have highlighted the significance of constructing clinical prediction models based on retrospective clinical data to guide clinical practice (28). In this study, using single-center clinical data, we analyzed the clinical prediction model and identified the potential optimal range of values for DAPT during the perioperative period of unruptured aneurysm stent-assisted coil embolization. Through the analysis, we determined the cutoff range for the PAR index and simulated the ROC curve to identify the optimal range of values. These findings were then compared with the optimal cutoff values for each PAR to finalize the final optimal range of PAR values. Although the number of positive cases of ischemic events was relatively small and the data were limited to single-center samples, which may introduce have errors, the results are consistent with the clinical reality observed in our center, providing meaningful guidance for clinical medication.

It is worth noting that Kan et al. (36) suggested that aneurysm size is a key factor in adjusting antiplatelet therapy dosage. Larger aneurysms are typically associated with stronger hemodynamic changes, which increase the risk of thrombosis and may therefore require higher doses of antiplatelet drugs or stronger medications. Furthermore, the study includes both ischemic and hemorrhagic events, but only mentions puncture site bleeding and does not address intracranial hemorrhage. In clinical practice, puncture site bleeding is closely related to puncture technique, operator experience, and postoperative compression measures. Therefore, we believe that puncture site bleeding should not be classified as a hemorrhagic complication related to DAPT. Consistent with this study, no cases of intracranial hemorrhage were observed in our research, and therefore hemorrhagic complications were not addressed. However, our study did not consider the impact of aneurysm size on antiplatelet drug dosing, which is a limitation of our study.

Based on genetic polymorphism of *CYP2C19*, which affects the efficacy of clopidogrel (21, 22, 43, 44), this study excluded the influence of *CYP2C19* genetic polymorphism on PAR during DAPT (aspirin + clopidogrel), by incorporating *CYP2C19* genotyping. Patients identified as intermediate or poor metabolizer were switched from clopidogrel to ticagrelor (90 mg twice daily) and excluded from the study (19). The CHANCE2 large-scale clinical trial has demonstrated that in carriers of *CYP2C19* loss-of-function alleles, DAPT with ticagrelor and aspirin is more effective in reducing the ischemic event recurrence in patients with cerebral microcirculation disorders compared to DAPT with clopidogrel and aspirin (19). Therefore, the data from patients excluded due to *CYP2C19* genotyping in our center merit further research and discussion.

Endovascular SAE for UIAs is also associated with many other complications, such as postoperative aneurysm re-rupture and delayed postoperative hemorrhage (45, 46). The DAPT used can also lead to gastric mucosa irritation, so concomitant gastroprotective therapy is often recommended. Some patients use proton pump inhibitor omeprazole as gastroprotective medication (47). However, studies have indicated that concomitant use of omeprazole with clopidogrel may affect latter’s platelet anti-aggregation ability, potentially introduce errors into our results, warranting further investigation and confirmation (47, 48). In contrast, in our study, in which all patients were treated with lansoprazole, it was shown that the main metabolizing enzyme of lansoprazole is *CYP3A4*, which does not compete with *CYP2C19*, and has a lesser impact on the efficacy of clopidogrel (49).

### Limitation

This study has several limitations. First, it is a single-center retrospective study, and the data may be subject to bias. Second, the study mainly focused on ischemic events and did not consider other hemorrhagic complications, apart from intracranial hemorrhage, which may introduce bias when assessing the benefits of antiplatelet therapy. Furthermore, the study did not perform post-thromboembolic event PAR re-testing, so the dynamic changes in platelet aggregation after complications could not be assessed. Additionally, the exclusion of PPI from the study may introduce bias. Future research should continue to collect clinical data from our center and further investigate these issues.

## Conclusion

In conclusion, our study provides single-center, large-sample clinical data on the use of DAPT during the perioperative period of SAE for UIAs and its association with the occurrence of ischemic events. We identified the clinical significance of PAR in guiding perioperative DAPT and established a range of PAR indicators. Additionally, we constructed a clinical prediction model, which contributes to the application in clinical practice.

## Data Availability

The raw data supporting the conclusions of this article will be made available by the authors, without undue reservation.
